# Impact of cultural intelligence on the cross-cultural adaptation of international students in China: The mediating effect of psychological resilience

**DOI:** 10.3389/fpsyg.2023.1077424

**Published:** 2023-03-14

**Authors:** Kequn Chu, Fengshu Zhu

**Affiliations:** ^1^College of Educational Science, Guangxi Science and Technology Normal University, Laibin, China; ^2^College of Physical Education, Yangzhou University, Yangzhou, China

**Keywords:** cross-cultural adaptation, cultural intelligence, psychological resilience, international students in China, mental health

## Abstract

**Introduction:**

Cultural intelligence can affect the cross-cultural adaptation of international students in China, but the mechanism of its influence is still unclear. This study examines the mediating effect of the psychological resilience of international students in China in the process of cultural intelligence affecting cross-cultural adaptation. We used the cultural intelligence scale, psychological resilience scale, and cross-cultural adaptation scale to measure 624 foreign students in China.

**Results:**

(1) There is a significant positive correlation between the cultural intelligence, psychological resilience, and cross-cultural adaptation of international students in China. (2) Resilience plays a mediating effect in the influence of the cultural intelligence of international students in China on cross-cultural adaptation.

**Conclusion:**

The cultural intelligence of international students in China can directly affect their cross-cultural adaptation and can also affect their cross-cultural adaptation through the mediating effect of psychological resilience.

## Introduction

As increasing numbers of foreign students choose to study and live in China, under the influence of foreign cultures, international students in China will suffer from maladjustment (Traore and Diarra, 2019). However, research on the adaptation of foreign students is still lacking, and the existing research in this area is mostly focused on Western developed countries (Major, 2005; Hu and Jiang, 2021). Due to differences in culture and national conditions, foreign research results cannot fully explain the living conditions of international students in China. Therefore, it is particularly necessary to carry out research on the adaptation of international students in China. This study explores the influence mechanism of cultural intelligence on the intercultural adaptation of international students in China, and these results have a certain application value.

Cross-cultural adaptation refers to a conscious and inclined behavior choice and behavior adjustment made by individuals based on their cognitive and emotional attachments to two cultures after they have transferred from one culture to another (Nesdale and Mak, 2000). Cross-cultural adaptation includes six main dimensions: environmental adaptation, daily life adaptation, language adaptation, communication adaptation, learning adaptation, and psychological adaptation (Malik et al., 2014). The level of cross-cultural adaptation directly determines whether individuals can adapt to foreign cultures and their living conditions in foreign cultures. Cross-cultural adaptation can be seen as both a result and a process, which is inevitably affected by the internal resources and capabilities of individuals. Individuals have significant differences in cross-cultural adaptation. Previous studies have shown that individual personality traits, social perception, stereotypes, ethnocentrism, and cultural intelligence all affect an individual's cross-cultural adaptation (Berry, 1998; Moon, 2010; Harrison, 2012; Lin et al., 2012; You and Dyne, 2012). Among them, more and more researchers have paid attention to cultural intelligence because of its significant space for adjustment and improvement.

The theory of cultural intelligence dictates that cultural intelligence is the ability of an individual to collect and process information, make judgments, take appropriate effective measures, and mobilize their own psychological energy to adapt to the new culture in the new cultural background (Earley and Ang, 2003). Researchers generally divide cultural intelligence into four dimensions: metacognitive cultural intelligence, cognitive cultural intelligence, motivational cultural intelligence, and behavioral cultural intelligence (Ang et al., 2007). Among these, metacognitive cultural intelligence is a psychological ability to think about the personal thinking process, predict other people's cultural preferences, and adjust psychological models during and after the cross-cultural experience (Flavell, 1979). Cognitive cultural intelligence reflects knowledge of the norms, practices, and conventions in different cultures acquired from education and personal experiences (Brislin et al., 2006). Motivational cultural intelligence reflects the ability to direct attention and energy toward learning about and functioning in situations characterized by cultural differences (Bandura, 2002). Behavioral cultural intelligence is the ability to exhibit appropriate verbal and non-verbal actions in culturally diverse situations (Gudykunst et al., 1988; Ang et al., 2007). Studies have shown that the level of cultural intelligence can significantly predict cross-cultural adaptation (Lin et al., 2012; Hu et al., 2020). Individuals with high levels of cultural intelligence have stronger motivation levels to adapt to foreign cultures and are more inclined to change the behavior patterns used in their original culture to adapt to the requirements of the new cultural environment, while individuals with low cultural intelligence appear passive when adapting to foreign cultures (Lin et al., 2012). However, the psychological mechanism of the influence of cultural intelligence on cross-cultural adaptation is still unclear and needs further exploration.

Psychological resilience is the ability of an individual to effectively adapt to life adversity such as stress, frustration, and trauma, which could help them to maintain good functions, even under pressure, with the positive energy of the individual (Xie et al., 2014). This positive energy is closely related to cross-cultural adaptation and adaptation to the new environment. It has been found that psychological resilience is positively correlated with cross-cultural adaptation (Sit et al., 2017). Low levels of psychological resilience are an important factor in cross-cultural maladjustment (Reichard et al., 2014). Psychological resilience enables individuals to withstand more destructive environmental changes, produce as few adverse behavior reactions as possible, recover from negative experiences, and flexibly adapt to the external environment (Masten and Cicchetti, 2016). One study also found that psychological resilience is a kind of positive psychological energy that drives individuals to actively adapt and make changes but requires certain arousal conditions, which mainly include risk factors and protective factors. These protective factors include individuals' positive personality traits, while cultural intelligence includes the collection and processing of information to make judgments. To mobilize the characteristics and abilities of their own psychological positive energy, individuals with higher cultural intelligence are more likely to mobilize their own psychological functions to achieve a suitable stress state to adapt to the new environment (Kumpfer, 2002). Therefore, this study investigates the relationship between cultural intelligence, psychological resilience, and cross-cultural adaptation, with international students in China as the research objects. Based on existing theoretical and empirical research, the following hypotheses are put forward: (1) Cultural intelligence significantly positively predicts the cross-cultural adaptation of international students in China. The higher the cultural IQ, the stronger the cross-cultural adaptability. (2) There is a significant positive correlation between cultural intelligence and psychological resilience among international students in China. (3) There is a significant positive correlation between psychological resilience and cross-cultural adaptation among international students in China. (4) The cultural intelligence of international students in China influences cross-cultural adaptation through psychological resilience; that is, psychological resilience plays a mediating effect between cultural intelligence and cross-cultural adaptation.

## Methods

### Participants

In this study, we selected 772 international students in China by random sampling from 26 universities in Beijing, Shanghai, Guangxi, and other provinces in China for a questionnaire survey. These questionnaires were all in English. Before the survey, the researchers had confirmed that the participants could understand all items. In this study, we distributed 772 questionnaires and 664 valid responses. After eliminating the questionnaires with wrong answers, serious data loss, and obvious answering rules, 624 valid questionnaires were finally obtained, with an effective rate of 81%. The demographic characteristics of the sample are given in Table 1.

**Table 1:** Demographic characteristics of the sample.

**Demographic variables**		**Numbers**
Gender	Men	314
	Women	310
Age	< 20	120
	20–25	396
	26–30	48
	31–35	26
	36–40	12
	>40	22
Education	Students in school	164
	Undergraduates	344
	Masters	82
	Doctors	18
	Others	16
Time in China	1–6 months	338
	7–12 months	122
	13–24 months	82
	25–36 months	34
	>36 months	48
Nationality	South Korea	152
	France	80
	United States	68
	Kazakhstan	54
	Russia	40
	Italy	26
	Others	204

## Measures

### Cultural intelligence

We used the English version of the cultural intelligence scale (CQS) to assess cultural intelligence. The CQS has 20 questions, including four dimensions: metacognition, cognition, motivation, and behavior (Ang et al., 2007). The higher the score, the higher the cultural and intellectual level. Cronbach's α of the scale in this study is 0.91. We used Amos 22.0 to perform confirmatory factor analysis on the scale, and the model fitting results showed that χ^2^/df = 2.15, CFI = 0.93, TLI = 0.92, IFI = 0.93, and RMSEA = 0.06. These results prove that the scale has adequate reliability and validity.

### Psychological resilience scale

We used the English version of the psychological resilience scale to assess psychological resilience. The scale has 25 questions in total, including five dimensions: ability, instinct, acceptance of change, control, and spiritual influence (Connor and Davidson, 2003). The higher the score, the higher the level of psychological resilience. Cronbach's α of the scale in this study is 0.85. We used Amos 22.0 to perform confirmatory factor analysis on the scale, and the model fitting results showed that χ^2^/df = 2.69, CFI = 0.89, GFI = 0.94, IFI = 0.89, and RMSEA = 0.07. These results prove that the scale has adequate reliability and validity.

### Cross-cultural adaptation

We used the English version of the cross-cultural adaptation scale to assess cross-cultural adaptation. The scale has 17 items in total, including six dimensions of environmental adaptation, daily life adaptation, language adaptation, communication adaptation, learning adaptation, and psychological adaptation (Furnham and Bochner, 1986). The higher the score, the higher the level of cross-cultural adaptation. Cronbach's α of the scale in this study is 0.83. We used Amos 22.0 to perform confirmatory factor analysis on the scale, and the model fitting results showed that χ^2^/df = 2.39, CFI = 0.88, GFI = 0.92, IFI = 0.89, and RMSEA = 0.07. These results prove that the scale has adequate reliability and validity.

## Data analysis

SPSS 23.0 was used for correlation analysis, and Amos 22.0 was used for mediating effect analysis of psychological resilience.

## Results

### Description of statistical analysis

In this study, we first performed descriptive statistical tests on each variable and its internal dimensions, and the results are shown in Table 2. The average cultural intelligence of international students in China reached 4.78 ± 0.89, indicating that the cultural intelligence level of international students in China was relatively high. The average psychological resilience of international students in China was 3.86 ± 0.55, indicating that the level of psychological resilience was at the middle level. The average value of cross-cultural adaptation of international students in China is 3.41 ± 0.59, indicating that the overall level of cross-cultural adaptation of international students in China was average.

**Table 2:** Descriptive statistics of variables and dimensions (*n* = 624, M ± SD).

	**M**	**SD**		**M**	**SD**
Metacognition CQ	4.9	1.17	Spiritual influence	3.69	0.93
Cognition CQ	4.28	1.14	Psychology resilience	3.86	0.55
Motivation CQ	5.34	1.09	Environmental adaptation	3.24	0.91
Behavior CQ	4.72	1.16	Daily life adaptation	3.3	0.82
Cultural intelligence	4.78	0.89	Language adaptation	3.14	1
Ability	4.01	0.62	Communication adaptation	3.45	0.82
Instinct	3.74	0.63	Learning adaptation	3.12	0.73
Accept change	3.82	0.67	Psychological adaptation	3.62	0.86
Control	3.9	0.72	Cross-cultural adaptation	3.41	0.59

The correlation analysis is used to explore the relationship between the variables, as shown in Table 3. It can be seen from Table 3 that, consistent with the research hypothesis, cultural intelligence and its four dimensions have a significant positive correlation with psychological resilience and a significant positive correlation with cross-cultural adaptation and the correlation between cultural intelligence as a whole and resilience; cross-cultural adaptation is greater than that between each subdimension of cultural intelligence, psychological resilience, and cross-cultural adaptation. There is a significant positive correlation between psychological resilience and cross-cultural adaptation.

**Table 3:** Correlation analysis between cultural intelligence, psychological resilience, and cross-cultural adaptation.

	**1**	**2**	**3**	**4**	**5**	**6**
1. Metacognition						
2. Cognition	0.59^**^					
3. Motivation	0.55^**^	0.35^**^				
4. Behavior	0.59^**^	0.42^**^	0.43^**^			
5. Cultural intelligence	0.85^**^	0.78^**^	0.73^**^	0.77^**^		
6. psychological Resilience	0.51^**^	0.40^**^	0.47^**^	0.45^**^	0.57^**^	
7. Cross-cultural adaptation	0.40^**^	0.42^**^	0.38^**^	0.36^**^	0.50^**^	0.56^**^

^**^p < 0.01, ^***^p < 0.001.

### The mediating effect of psychological resilience

**Figure 1:**
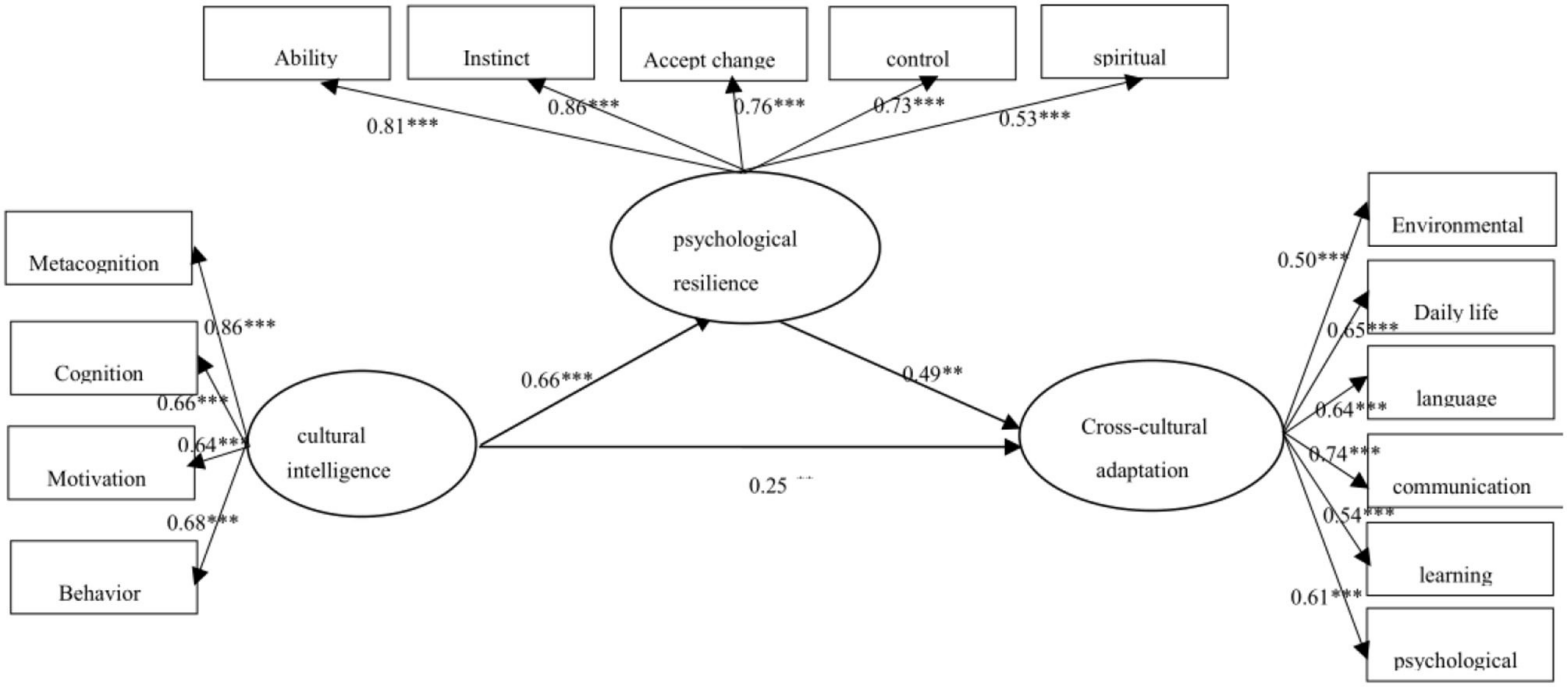
Mediation effect model. Note. ^**^p < 0.01, ^***^p < 0.001.

Based on correlation analysis, we used the structural equation model (SEM) to explore the mechanism of cultural intelligence affecting cross-cultural adaptation, and the maximum likelihood method is used to estimate and test it. The results are as follows: χ^2^ = 247.18, df = 87, χ^2^/df = 2.84, NFI = 0.88, CFI = 0.92, IFI = 0.92, and RMSEA = 0.06, indicating that the overall model fit is adequate. Further study on the parameter estimation of the model shows that the path coefficients in the model have reached a significant level, and the results are shown in Figure 1. It can be seen from Figure 1 that the direct effect of cultural intelligence on cross-cultural adaptation is significant, the path coefficient of cultural intelligence to psychological resilience is significant, and the path coefficient of psychological resilience to cross-cultural adaptation is also significant. Therefore, psychological resilience plays a part in the mediating effect between cultural intelligence and cross-cultural adaptation. Through the analysis of the effect size, it was found that the standardized direct effect accounted for 43.60% of the total effect. The indirect effect accounted for 56.40% of the total effect.

## Discussion

The purpose of this study was to explore the influence mechanism of cultural intelligence on the cross-cultural adaptation of international students in China. The results of this study show that the overall level of cross-cultural adaptation of international students in China is low, and there is a positive correlation between cultural intelligence and cross-cultural adaptation among international students in China, which is consistent with the previous study (Lin et al., 2012; Chen et al., 2014; Setti et al., 2020). International students in China should have a higher level of cultural intelligence to better adapt to the Chinese culture that is unfamiliar to them. As far as the relevance of specific dimensions of cultural intelligence to cross-cultural adaptation is concerned, cognitive cultural intelligence has the most prominent impact on cross-cultural adaptation, which indicates that the higher the cognitive cultural intelligence of international students in China, the more inclined they are to learn and follow the rules of Chinese culture when adapting to Chinese culture and the better they integrate into Chinese culture. The results also showed that the cultural intelligence of international students in China had a positive predictive effect on their psychological resilience, that is, the higher the level of cultural intelligence of international students in China, the stronger their psychological resilience. On the contrary, the lower the level of cultural intelligence, the weaker the psychological resilience of international students in China. As an internal resource for individuals, psychological resilience is not only affected by cultural intelligence but also can be improved through training. In addition, metacognitive cultural intelligence has the strongest correlation with psychological resilience, indicating that international students in China with higher metacognitive cultural intelligence are more inclined to take active and effective measures to change their original cultural habits in cognition to better adapt to the rules and habits of new and different cultures when encountering adaptation difficulties.

The study also found that cultural intelligence has an impact on cross-cultural adaptation through the mediating effect of psychological resilience. This is consistent with previous research results (Kumpfer, 2002; Bücker et al., 2014). Some studies have shown that psychological resilience has significant positive predictability for individuals to actively adapt to a new environment (Kumpfer, 2002; Bücker et al., 2014). Psychological resilience is a kind of positive psychological resource. When an individual faces a source of stress, psychological resilience can help the individual to better cope with it, thus producing a lower stress response. This conclusion is also reflected in the group of foreign students. Psychological resilience is an important influencing variable of cross-cultural adaptation, and high psychological resilience will enhance the cross-cultural adaptation of international students in China. On the contrary, when psychological resilience is relatively low, cross-cultural adaptation will be relatively low. Cultural intelligence can enhance cross-cultural adaptation by enhancing psychological resilience. In addition, the indirect effect represented by this mediating effect is large. From a numerical point of view, the standardized indirect effect accounts for 43.60% of the total effect, while the standardized direct effect accounts for 56.40%, indicating that the indirect effect and the direct effect are equally important. This shows that psychological resilience is an important mediating variable between cultural intelligence and cross-cultural adaptation.

### Strengths and limitations

In terms of theory, this study reveals the mechanism of cultural intelligence on cross-cultural adaptation and the applicability of the psychological resilience model supported by empirical evidence in explaining the relationship between cultural intelligence and cross-cultural adaptation. The results of this study also have a certain application value. This study has found that cultural intelligence can improve the psychological resilience of international students in China and promote their cross-cultural adaptation.

However, this study has the following limitations: first, the study has highlighted that the micro-intervention model based on cultural intelligence can effectively improve cultural intelligence (Reichard et al., 2014). Another result also verified the effectiveness of this model in improving the cultural intelligence of enterprise employees in Chinese culture (Zhang, 2013). Our study did not consider the effect of the intervention on the cross-cultural adaptation of international students in China. Therefore, future research should consider the intervention of cultural intelligence in international students in China to enhance their psychological resilience and therefore enhance their cross-cultural adaptability. Second, there are also some methodological limitations, such as the lack of a power calculation preceding the sample recruitment and the lack of a multiple comparison correction. Third, we adopted the convenient sampling method, which means that the research samples lack representativeness to a certain extent. Therefore, more scientific sampling methods should be selected for future research. Finally, we adopted the self-report method and used a cross-sectional design, which excluded our ability to determine causality. Future researchers could adopt a longitudinal method to confirm the influence mechanism linking cultural intelligence and cross-cultural adaptation.

## Data availability statement

The raw data supporting the conclusions of this article will be made available by the authors, without undue reservation.

## Ethics statement

The studies involving human participants were reviewed and approved by Academic Committee of Guangxi Science and Technology Normal University. The participants provided their written informed consent to participate in this study.

## Author contributions

KC is responsible for questionnaire distribution and data processing and paper writing. FZ is responsible for revising project management papers. Both authors contributed to the article and approved the submitted version.
